# Trends and Factors Associated With Ventilator-Associated Pneumonia: A National Perspective

**DOI:** 10.7759/cureus.23634

**Published:** 2022-03-29

**Authors:** Harshil Shah, Ahmed Ali, Achint A Patel, Vaidarshi Abbagoni, Ruchir Goswami, Ananth Kumar, Felipe Velasquez Botero, Elohor Otite, Hardik Tomar, Maheshkumar Desai, Prakash Maiyani, Hiteshkumar Devani, Faraz Siddiqui, Salman Muddassir

**Affiliations:** 1 Hospital Medicine, Guthrie Robert Packer Hospital, Sayre, USA; 2 Internal Medicine, Al-Azhar University Faculty of Medicine for Boys, Cairo, EGY; 3 Internal Medicine, Oak Hill Hospital, Brooksville, USA; 4 Internal Medicine, MediCiti Institute of Medical Sciences, Hyderabad, IND; 5 Epidemiology and Public Health, Icahn School of Medicine at Mount Sinai, New York, USA; 6 Medicine, Sri Ramachandra Institute of Higher Education and Research, Chennai, IND; 7 General Medicine, Larkin Community Hospital, Miami, USA; 8 Psychiatry, Raleigh General Hospital, Beckley, USA; 9 Medicine, Kazan Federal University, Kazan, RUS; 10 Internal Medicine, Hamilton Medical Center, Medical College of Georgia/Augusta University, Dalton, USA; 11 Internal Medicine, Gold Coast University Hospital, Southport, AUS; 12 Dental Medicine, University of Pittsburgh School of Dental Medicine, Pittsburgh, USA; 13 Internal Medicine and Critical Care, Guthrie Robert Packer Hospital, Sayre, USA

**Keywords:** predictors, outcomes, trends, mechanical ventilation, ventilation associated pneumonia

## Abstract

Background: Ventilator-associated pneumonia (VAP) is a hospital-acquired pneumonia that occurs more than 48 hours after mechanical ventilation. Studies showing temporal trends, predictors, and outcomes of VAP are very limited.

Objective: We used the National database to delineate the trends and predictors of VAP from 2009 to 2017.

Methods: We analyzed data from the Nationwide Inpatient Sample (NIS) for adult hospitalizations who received mechanical ventilation (MV) by using ICD-9/10-CM procedures codes. We excluded hospitalizations with length of stay (LOS) less than two days. VAP and other diagnoses of interest were identified by ICD-9/10-CM diagnosis codes. We then utilized the Cochran Armitage trend test and multivariate survey logistic regression models to analyze the data.

Results: Out of a total of 5,155,068 hospitalizations who received mechanical ventilation, 93,432 (1.81%) developed VAP. Incidence of VAP decreased from 20/1000 in 2008 to 17/1000 in 2017 with a 5% decrease. Patients who developed VAP had lower mean age (59 vs 61; p<0.001) and higher LOS (25 days vs. 12 days; p<0.001). In multivariable regression analysis, we identified that males, African Americans, teaching hospitals and co-morbidities like neurological disorders, pulmonary circulation disorders and electrolyte disorders are associated with the increased odds of developing VAP. VAP was also associated with higher rates of discharge to facilities and increased LOS.

Conclusion: Our study identified the trends along with the risk predictors of VAP in MV patients. Our goal is to lay the foundation for further in-depth analysis of this trend for better risk stratification and development of preventive strategies to reduce the incidence of VAP among MV patients.

## Introduction

Ventilator-associated pneumonia (VAP) is defined as a lower respiratory tract infection that develops after 48 hours of mechanical ventilation (MV) [1]. Approximately 9% to 27% of all mechanically ventilated patients are susceptible to VAP and the risk is more predominant in the first five days [2]. A survey by the National Healthcare Safety Network (NHSE) has reported that VAP incidence for various types of hospital units is 0.0 to 4.4 per 1000 ventilator days [3].

Despite the major advances in diagnostic techniques and management, the morbidity and mortality rates related to VAP remain high [1]. In various studies, mortality rates ranged from 20% to 75% [1,4]. In the United States, this rate reached up to 13% in 2016 according to the Infectious Diseases Society of America (IDSA). One of the biggest challenges in the management of VAP includes the missing of a gold standard for diagnosis, the absence of effective preventative strategies, and the rise in antibiotic resistance [5]. Specific risk factors include the age of the patient, the presence of underlying chronic diseases, altered mental status, the length of hospital stay, duration of MV, the time between intubation-ICU admission, and the time to start the optimal antibiotic therapy [6]. Regarding the VAP financial burden, a study previously showed more than $40,000 USD increase of hospital charges per patients ($104,983 USD +/- $91,080 USD vs $63,689 USD+/- $75,030 USD, p < 0.001) [7]. Another study estimated that patients who develop VAP incur >/= $10,019 USD in additional hospital costs [8].

A lack of updated national studies is creating a large knowledge gap in the prevention and treatment of VAP, which is being reflected in the poor outcomes in the evolution of patients in the ICU. Therefore, to reduce the incidence of VAP in the United States and improve outcomes for critically ill patients, it is necessary to identify the current population prevalence of VAP and the risk factors associated with it. In this study, our objective is to retrospectively analyze a large national database from 2007 to 2017 to identify the current incidence, prevalence, risk factors, and outcomes in adult patients with VAP to help reduce associated morbidity and mortality.

## Materials and methods

Data source

The study cohort was derived from the National (Nationwide) Inpatient Sample (NIS) of the Healthcare Cost and Utilization Project (HCUP), Agency for Healthcare Research and Quality (AHRQ). NIS is one of the largest all-payer publicly available databases on inpatient discharges from U.S. hospitals maintained by the AHRQ. The NIS approximates a 20% stratified sample of discharges from U.S. community hospitals, excluding rehabilitation and long-term acute care hospitals and contains more than 7 million hospitalizations annually [9]. With the established weights in NIS, this data could be weighted to represent the standardized U.S. population and obtain national estimates with high accuracy [10].

Study population and design

We queried the 2009-2017 NIS database using International Classification of Diseases, 9th Revision, Clinical Modification and International Classification of Diseases, 10th Revision, Clinical Modification (ICD-9/10-CM) procedural codes for MV. These codes have been used by previously published articles from administrative databases such as NIS [11-15]. We excluded hospitalizations with length of stay (LOS) less than two days and those admitted due to primary diagnosis of community-acquired pneumonia (CAP). VAP was then identified with the presence of ICD-9/10-CM codes in secondary diagnostic fields.

We extracted demographics, hospital-level characteristics (geographical region, size, and teaching status) and patient-level characteristics. Detailed description of data elements of NIS databases is available on the HCUP website [16]. We estimated comorbidities using Elixhauser comorbidity software and mortality risk using the validated All Patient Refined Diagnosis Related Groups (APR-DRGs) mortality score, which are also supplied by HCUP tools and software [17,18]. We then further identified specific medical conditions and procedures of interest by using ICD-9/10-CM diagnosis and procedure codes.

Statistical analysis

We calculated the yearly proportion of VAP among the total cohort of adult hospitalizations requiring MV for more than two days to establish the incidence trend. We used regression model with year as independent predictor to analyze the temporal trends. Descriptive statistics were performed to present the baseline difference in sociodemographic, comorbidities and hospital level characteristics among those who developed VAP and those did not. We compared categorical variables with the chi-square test, and continuous variables with student's t-test or Wilcoxon rank-sum test depending on weather they are normally distributed or not. To estimate the impact of VAP on outcomes, we used logistic regression models (in-hospital mortality and discharge to a long-term facility) and adjusted them for potential confounders and APRDRG risk of mortality score. SAS 9.4 (SAS Institute, Cary, NC, USA) was used for all analyses. We performed all statistics using designated weight values to produce nationally representative estimates [10]. We used survey procedures were used where applicable to account for the inherent survey design of NIS to produce more robust estimates [19]. Two-tailed p-value <0.05 was considered to determine statistical significance.

## Results

We analyzed a total of 5,155,068 hospitalizations requiring more than two days of MV from 2008 to 2017. Out of total MV patients at risk, 93,432 (1.81%) developed VAP.

Temporal trends of VAP in mechanically ventilated patients

**Figure 1:**
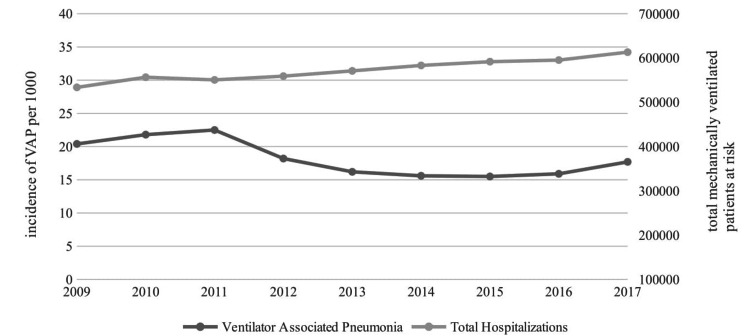
Incidence of ventilator-associated pneumonia in mechanically ventilated patients

The VAP incidence among intermittent mandatory ventilation (IMV) patients showed an initial increase between 2009 and 2011 from 2.04% in 2009 to 2.25% in 2011; after that the VAP incidence declined to 1.77% in 2017 with an overall decline of 4% yearly (OR 0.96, 95%CI: 0.94-0.98, p:0.001) (Figure 1).

Baseline characteristics of the study cohort

In our cohort, patients older than 65 years were the most prevalent age group developing VAP with 40.9%, followed by age group 50-64 with 31.9%. Patients who developed VAP were more likely to be male gender (62.4% vs 54.1%, p:0.001). Most patients who developed VAP were Caucasian (54.1%) followed by African American (18.5%; p:0.001). The most prevalent comorbidities among MV patients with VAP were fluid and electrolyte disorders at 64.9%, hypertension at 48.8%, weight loss at 29.2%, anemia at 27.3%, history of chronic pulmonary disease at 22.9%, congestive heart failure 21%, and other neurological disorders at 20.2%. Large bed size hospitals (71.1% vs 63.7%; p:0.001) and teaching hospitals (79.1% vs 64.3%; p:0.001) had higher proportion of VAP. Detailed description of baseline characteristics of the study cohort is depicted in Table 1. 

**Table 1:** Baseline characteristics of ventilator-associated pneumonia (VAP) in mechanical ventilation patients

Patient Characteristics	Patients without VAP	Patients with VAP	Total	p-value
Overall	5,061,636	93,432	5,155,068	
Age in years (%)				<0.001
18-34	9.7	12.1	9.7	
35-49	13.7	15.1	13.8	
50-64	30.4	31.9	30.4	
65-79	31.0	29.7	30.9	
>=80	15.2	11.2	15.2	
Gender (%)				<0.001
Male	54.1	62.4	54.2	
Female	45.9	37.6	45.8	
Race (%)				<0.001
White	62.2	54.1	62.0	
Black	16.2	18.5	16.2	
Hispanic	8.7	10.3	8.7	
Others	6.0	7.4	6.1	
Missing	7.0	9.8	7.0	
Comorbidities (%)				
Obesity	15.8	13.7	15.8	<0.001
Hypertension	56.4	48.8	56.3	<0.001
Diabetes mellitus with chronic complications	9.7	8.1	9.6	<0.001
diabetes mellitus without chronic complications	20.6	18.5	20.5	<0.001
Congestive heart failure	20.1	21.0	20.1	<0.001
Valvular heart disease	5.5	4.5	5.5	<0.001
History of chronic pulmonary disease	27.9	22.9	27.8	<0.001
Pulmonary circulatory disease	5.1	6.9	5.1	<0.001
Peripheral vascular disease	9.1	7.9	9.1	<0.001
Paralysis	7.5	16.5	7.6	<0.001
Coagulopathy	16.7	18.5	16.7	<0.001
solid tumor without metastasis	2.7	1.8	2.6	<0.001
lymphoma	0.9	0.8	0.9	<0.001
Metastatic cancer	3.2	1.9	3.2	<0.001
Weightloss	15.7	29.2	15.9	<0.001
Liver disease	6.2	5.5	6.2	<0.001
Alcoholism	11.3	9.4	11.2	0.767
other neurological disorders	15.8	20.2	15.9	<0.001
renal failure	20.2	17.2	20.1	<0.001
hypothyroidism	11.0	8.0	10.9	<0.001
arthritis	2.6	1.9	2.6	<0.001
anemia deficiency	25.3	27.3	25.3	<0.001
fluid and electrolyte disorders	58.6	64.9	58.7	<0.001
depression	11.5	8.1	11.4	<0.001
psychoses	7.8	4.7	7.7	<0.001
Drug abuse	8.0	5.0	7.9	<0.001
Median house hold income (%)				<0.001
1st quartile	31.7	30.8	31.7	
2nd quartile	25.4	24.7	25.4	
3rd quartile	22.6	23.7	22.7	
4th quartile	17.8	18.2	17.8	
Primary Insurance (%)				<0.001
Medicare/Medicaid	69.2	68.1	69.2	
Private including Health Maintenance Organization	20.9	22.7	20.9	
Uninsured/Self-pay	9.7	9.0	9.7	
Hospital bed size (%)				<0.001
Small	10.6	8.0	10.6	
Medium	25.2	20.0	25.1	
Large	63.7	71.1	63.8	
Hospital Type (%)				<0.001
Rural	5.8	2.9	5.8	
Urban-Non teaching	29.4	17.1	29.2	
Teaching	64.2	79.1	64.5	
Hospital region (%)				<0.001
Northeast	18.6	21.4	18.6	
Midwest	22.0	21.8	22.0	
South	39.3	33.8	39.2	
West	20.1	23.0	20.1	
Day of admission				<0.001
week day	74.5	73.7	74.5	
weekend	25.5	26.3	25.6	
Source of admission (%)				<0.001
Transfer from other hospital or other health facility	28.3	33.4	28.4	
Emergency department	71.7	66.6	71.6	
Type of admission (%)				0.094
Emergent or Urgent	89.9	89.7	89.9	
Elective	10.1	10.3	10.1	
Disposition status (%)				<0.001
Home	38.2	19.3	37.9	
Facility	37.5	61.5	38.0	
Died	24.1	19.0	24.0	
Length of stay (LOS) (mean±SE)	13 (±0.1)	25 (±0.3)		<0.001

Predictors of VAP incidence in mechanically ventilated patients

Our analysis identified several predictors associated with increased odds of development of VAP in MV patients. These predictors included the patients’ age where the highest odds were found among the age group 18-34 (OR 1.36, 95%CI: 1.27-1.46, p:0.0001), male gender (OR 1.40, 95%CI: 1.35-1.45, p:0.0001), and Hispanic race (OR 1.25, 95%CI: 1.16-1.34, p:0.0001). Furthermore, we found comorbidities, such as paralysis (OR 2.12, 95%CI: 2.02-2.22, p:0.0001), weight loss (OR 2.04, 95%CI: 1.93-2.17, p:0.0001), pulmonary circulatory disease (OR 1.38, 95%CI: 1.29-1.48, p:0.0001), and other neurological disorders (OR 1.29, 95%CI: 1.24-1.35, p:0.0001) were significantly associated with higher VAP incidence. However, the Midwest region (OR 0.77; 95%CI: 0.68-0.88, p:0.0001) as compared to the Northeast, rural hospitals (OR 0.44; 95%CI: 0.36-0.52, p:0.0001) and urban non-teaching hospitals (OR 0.47; 95%CI: 0.42-0.52, p:0.0001) were associated with lower odds of VAP incidence. Other detailed factors associated with VAP in MV patients are listed in Table 2. 

**Table 2:** Predictors of ventilator-associated pneumonia in mechanical ventilation patients HMO=Health Maintenance Organization; LL=lower limit; UL=upper limit

Independent variable/ Characteristic	Odd Ratio (OR)	95% CI (LL)	95% CI (UL)	P value
Year	0.95	0.93	0.97	< .0001
Age in years				
18-34	1.36	1.27	1.46	< .0001
35-49	1.27	1.20	1.35	< .0001
50-64	1.22	1.17	1.27	< .0001
>=65	Ref			
Gender				
Male	1.40	1.35	1.45	< .0001
Female	Ref			
Race				
White	Ref			
Black	1.23	1.15	1.31	< .0001
Hispanic	1.25	1.16	1.34	< .0001
Others	1.24	1.15	1.34	< .0001
Comorbidities				
Obesity	0.99	0.94	1.04	0.6055
Hypertension	0.83	0.80	0.87	< .0001
Diabetes mellitus	0.90	0.84	0.95	0.0005
Congestive heart failure	1.22	1.17	1.28	< .0001
Anemia deficiency	1.17	1.12	1.22	< .0001
Pulmonary circulatory disease	1.38	1.29	1.48	< .0001
Paralysis	2.12	2.02	2.22	< .0001
Coagulopathy	1.03	0.98	1.08	0.2273
Weight loss	2.04	1.93	2.17	< .0001
Alcoholism	0.80	0.75	0.85	< .0001
Drug abuse	0.63	0.58	0.68	< .0001
Other neurological disorders	1.29	1.24	1.35	< .0001
Renal failure	0.84	0.81	0.89	< .0001
Electrolyte and fluid disorders	1.26	1.21	1.32	< .0001
Median household income				
1st quartile	0.97	0.90	1.05	0.4394
2nd quartile	1.02	0.96	1.10	0.5157
3rd quartile	1.05	0.99	1.11	0.1344
4th quartile	Ref			
Primary Insurance				
Medicare/Medicaid	Ref			
Private including HMO	0.98	0.93	1.03	0.3936
Uninsured/Self-pay	0.82	0.76	0.88	< .0001
Hospital region				
Northeast	Ref			
Midwest	0.77	0.68	0.88	< .0001
South	0.80	0.71	0.91	0.0005
West	1.07	0.94	1.21	0.3201
Hospital Type				
Rural	0.44	0.36	0.52	< .0001
Urban Non-teaching	0.47	0.42	0.52	< .0001
Teaching	Ref			
Hospital bed size				
Small	0.69	0.61	0.77	< .0001
Medium	0.75	0.68	0.83	< .0001
Large	Ref			

Outcomes of mechanically ventilated patients with VAP

**Figure 2:**
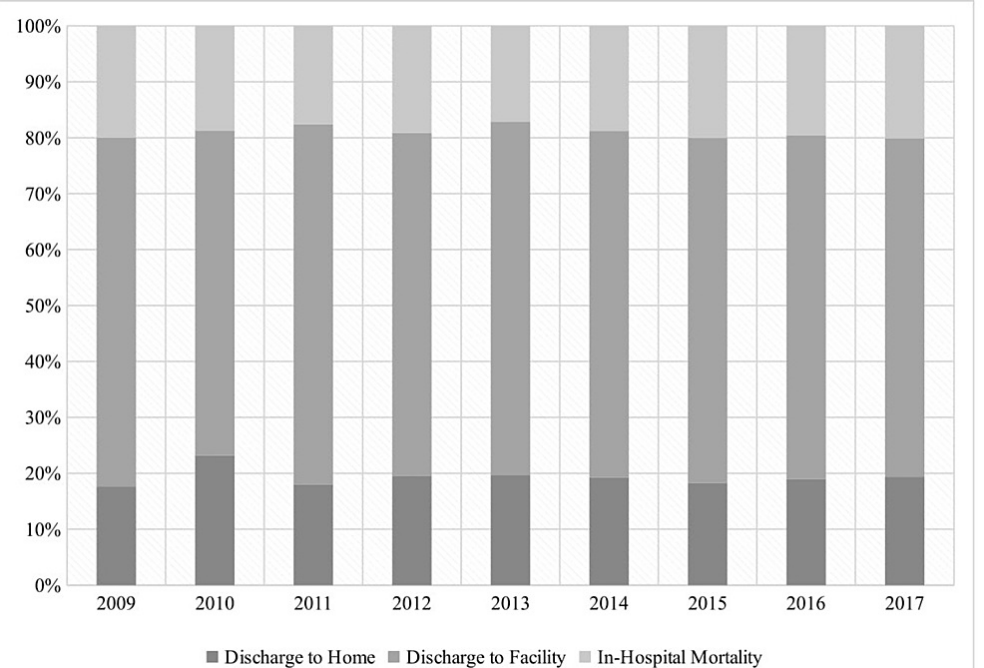
Temporal trends of discharge disposition among MV patients developing VAP MV=Mechanical Ventilation, VAP=Ventilator Associated Pneumonia

**Figure 3:**
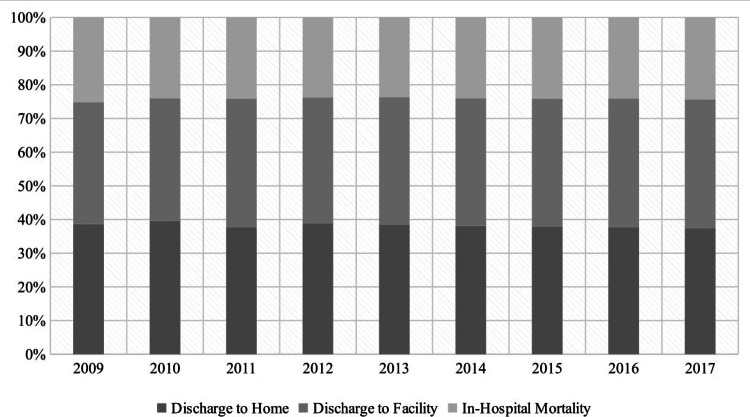
Temporal trends of discharge disposition among MV patients without VAP MV=Mechanical Ventilation, VAP=Ventilator Associated Pneumonia

**Figure 4:**
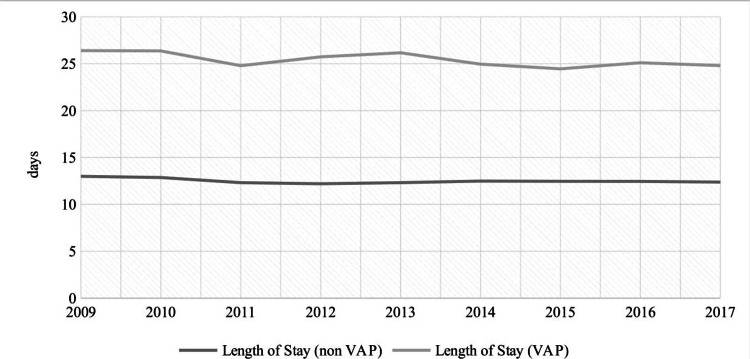
Temporal trends of length of stay in MV patients by VAP MV=Mechanical Ventilation, VAP=Ventilator Associated Pneumonia

MV patients who developed VAP had higher proportion of discharge to facilities (61.5% vs 37.6%; p:0.0001) but lower proportion of in-hospital mortality (19.0% vs 24.1%; p:0.0001). The mean LOS among VAP patients was significantly higher (25.4 ± 0.3 days vs 12.5 ± 0.05 days; p:0.0001) as compared to those who did not develop VAP. In trend analysis, discharge to facilities remained stable from 62.34% in 2009 to 60.56% in 2017 (p:0.067) among patients who developed VAP. We observed similar trend in case of in-hospital mortality, however, the mean LOS declined mildly from 26 days in 2009 to 24 days in 2017 (p:0.001) (Figures 2, 3, 4).

## Discussion

In the present study, a constant and progressive increase was found in the number of hospitalized patients who received MV between 2009 and 2017. Regarding the incidence of VAP in patients with MV, an increase in cases was recorded between the years 2009 to 2011. Interestingly, the incidence of VAP decreased considerably between 2011 and 2015, and then experienced a slight progressive increase towards 2017, but without reaching the levels of the 2009 - 2011 period. With these results, a clear variation in the incidence of VAP can be observed, which had not been registered in previous years. A study done on VAP in Minnesota reported a steady VAP incidence between 2003 and 2009 [20]. Another study done in Italy revealed a marked decrease of 9.2% in VAP incidence between 2004 and 2010 [21]. With advances in medicine during this period of time, it could be determined that there was an improvement in the prevention of VAP and/or the early management of patients who developed this infection, which would explain the changes in the incidence of patients with VAP. However, other factors that directly influence the results found in this study should be considered. VAP diagnosis could be subjective and nonspecific since there are no clinical, imaging, or laboratory criteria that universally define this condition, which could lead to an under-registration of cases of VAP among the different institutions across the US [22]. On the other hand, some institutions do not carry out a complete registry of hospital-acquired infections for financial reasons with different health insurers, leading to falsely lower VAP rates [20].

In our study, we found several significant independent predictors for VAP development in MV patients related to their age, gender, race, comorbidities, insurance status, and hospital region, size, and type. Young age was an independent predictor for VAP development in MV patients, contrary to the classic idea of elderly patients being at higher risk of VAP development as in Majumdar and Padiglione [23] or the idea of age being an insignificant predictor for VAP development as in Blot et al.'s study [24]. However, our analysis matches the outcomes of the 2018 French study by Dananché et al. [25]. We propose the rationale behind our findings is that young patients receive intubation more often for surgical etiologies (trauma or elective surgeries) rather than medical etiologies. In the 2010 Cook et al. study, VAP development odds ratio was four times more in trauma patients than non-trauma patients (OR 3.68, 95%CI 2.26-5.99, p:0.001) [26] and five times in the 1998 Cook et al. study (risk ratio 5.0, 95%CI 1.91-13.11) [27]. The 2010 Cook et al. study showed higher prevalence of young patients in trauma patients [26]. Unlike patients intubated for medical illnesses, trauma patients are more prone to contaminated environments and less preparation time, which may result in higher infection risks. Another rationale is combustible tobacco use, which is more prevalent in the younger population. In Cornelius et al., combustible tobacco use prevalence was the highest in the population aged 25-44 at 20.1% (19.1-21.1). On the other hand, the population older than 65 years had the least combustible tobacco use prevalence at 9.9% (9.2-10.6) [28]. Generally, tobacco smoking has adverse effects on the airways’ cilia function and alters the normal flora in the mouth. Nasiriani et al., which studied oral hygiene and VAP incidence, found a significant relationship between VAP incidence and smoking (p:0.001) [29]. Other possible risks for younger patients are non-compliance to medical advice, delayed medical care seeking, and maybe lower infection control precautions taken by the healthcare providers compared to the precautions taken for elderly patients. We recommend further research with consideration of these factors and stratification of the patients based on the causes of their ICU admission and their social history. Male gender was another significant independent predictor of VAP development; this finding is consistent with a previous study by Wałaszek et al. [30]. Male gender, like the patient’s age, is associated with higher surgical etiologies for intubation which result in higher VAP incidence [26]. Moreover, tobacco smoking prevalence in men was 20.1% (19.3-20.9), compared to 13.6% (12.9-14.3) in women [28]. It may be worth mentioning, despite the lower incidence of VAP in women, their VAP mortality rate is significantly higher with OR of 3.47 (95%CI: 1.25-9.66, p:0.017) [31]. Regarding race, most previous studies mentioned the patients’ races in the form of frequency without adjusted statistical analysis, and like in our study, the Caucasian race was usually the most prevalent race among the VAP-associated hospitalization [32]. With this lack of adjustment, we cannot establish the Caucasian race as a predictor of VAP development depending on the frequency only. Interestingly, our adjusted analysis found that the non-White races are significant independent predictors of VAP development in MV patients. The highest OR was found in the Hispanic population at 1.25 (95%CI: 1.16-1.34, p:0.0001). We propose several possible contributors like socioeconomic background, healthcare accessibility, causes of ICU admission, prevalence of chronic illnesses, and geographical distribution. Those possible contributors require more investigation within each race to reach the best explanation and develop preventive measures.

Regarding comorbidities, we found paralysis has the highest VAP OR, which is consistent with the 2010 Cook et al. study [26]. This also applies to the other neurological diseases which have lower, yet significant, OR for VAP development [24,27]. The lack of protective airways reflexes (cough) and unregulated mucosal secretions (vagal dysfunction) in those patients may contribute to lung infections. Moreover, those patients are prone to gastric reflux and aspirant pneumonias. Other significant predictors were pulmonary circulatory disease and congestive heart failure which are consistent with previous studies [24,27]. The inadequate circulation in these conditions adversely affects the lungs immunity. Pathogen clearance, immune cell delivery, and tissue oxygenation all need good lung circulation to function properly. Moreover, the chronic nature of these conditions results in irreversible tissue damage and decreases in the lung functional reserve. Moving forward to weight loss as a predictor, it was found to be a significant VAP predictor in Cook et al. [26]. Weight loss shows the patients’ frailty and their lesser ability to recover. In addition, it may reflect an underlying undiagnosed malignancy or infection. Those patients tend to be in an immunocompromised state and the risks of infections are high. Lastly, fluid and electrolytes disorders were the highest prevalent comorbidities between the VAP patients (64.9%) and they showed a significant predictor OR of 1.26 (95%CI: 1.21-1.32, p:0.0001), moreover, this significance was detected by the Cook et al. [26]. Whether the fluid and electrolyte disorders affect the VAP incidence through affecting the circulatory functions, the neurological functions, or both, further research is needed to illustrate the causality between those disorders and VAP development.

Our adjusted analysis showed a significant lower OR of VAP incidence in the uninsured/self-pay MV patients. We can assume that those patients are free from major illnesses (ineligible for Medicare), unemployed, and their income is above the threshold for Medicaid eligibility. In these situations, both patients and doctors are actively trying to minimize the length of ICU stay, which may have a positive effect on VAP incidence. In addition, they may undergo less extensive diagnostic procedures before ICU discharge, which may result in missed VAP diagnoses, and those patients are reluctant to revisit the hospital after developing symptoms because of the fear of extra expenses. Therefore, we hypothesize that those healthy patients have higher chances to survive the short ICU stay for their less complicated conditions with possibility of undiagnosed VAP cases. These assumptions need further testing and research focusing on the effect of the insurance status on the VAP incidence in MV patients. The Midwest and South regions' hospitals showed lower incidence of VAP infections compared to the Northeast and West regions' hospitals. The fact that almost all hospitals in the United States are following the same ICU guidelines makes us exclude the practice discrepancy from the possible causes. Similarly, the patients’ baseline characteristics were adjusted for sake of generalizability. This leaves us with the possibility of unmeasured confounding or effect modification. Possible contributors are the discrepancy of the ICU admission causes and the environmental factors. For example, air pollution (industrial or wildfires) is the worst in the West. Likewise, fungal respiratory infection (blastomycosis) is endemic in the Northeast. Further research is needed to eliminate the doubts regarding the VAP odds discrepancy.

When it comes to the hospitals’ size and type, our analysis found large-sized hospitals are a predictor for VAP development. This contradicts the Lee et al. study, which found small-sized hospitals being a predictor for VAP development [33]. They rationalized their findings by the relative unfamiliarity with the use of the ventilator, the relative inexperience with protocols for VAP prevention, and the shortage of skilled staff in the small-sized hospitals. However, their study included only 247 VAP cases from community-based hospitals. Considering that, our study found the teaching hospitals being significant independent predictors for VAP development, and those teaching hospitals are usually either large or medium hospitals, therefore, we assume that if they had included the teaching hospitals while investigating the hospitals’ size as a predictor for VAP development, this would have altered their results. Moreover, we can rationally anticipate higher odds of VAP development in both large hospitals and teaching hospitals for different reasons. For example, the medical service provided by those hospitals is usually more complex and directed toward trauma patients, cancer patients, organ transplant patients, and patients with one or more system failures. In addition, those patients are prone to longer and more complicated ICU stays including the risk of VAP development. In addition, the same study, Lee et al., found the ventilator utilization ratio (VUR) to be the highest in large hospitals. The VUR is defined as the proportion of ICU days being spent on invasive mechanical ventilation, and with the longer ventilator periods, VAP developments odds become higher [24,34]. Lastly, teaching hospitals may possess extra risks, as they are involved in physician training, new protocol development, and clinical trials, which may result in adverse outcomes and complications.

During the period 2009 to 2017, discharge to a facility continued to be the most common outcome among VAP patients with an average of 61.7%. To the best of our knowledge, the discharge disposition of VAP has not been studied before, at least on this scale. These results reflect the long-term encumbrance of VAP on the patients’ quality of life even after their discharge. Nonetheless, the significant economic burden is not only affecting the patients after their discharge but rather starting much earlier by extending their hospital length of stay. The average hospital length of stay in the MV patients who developed VAP was 25 days compared to 12 days in non-VAP patients. The Corrado et al. study showed similar results with median LOS of 22 days for VAP (IQR: 11-39 days), and the hospitalization lasted ≥14 days for 65.9% of the VAP patients [32]. Regarding the in-hospital mortality rates, our study showed an average of 19% mortality rate in VAP patients contrary to the previous studies which had mortality rates ranging from 30% to 60% [7,35]. This might falsely indicate improvement in VAP management; however, our study did not show significant mortality rates variations across the years despite the decrease in the VAP incidence, which signifies relative improvement in VAP prevention rather than VAP management. Moreover, the previous studies had smaller samples, which makes us question their results’ power. Likewise, the VAP discharge to home rates did not show significant improvements across the years. In order to improve VAP management, it is necessary to develop better diagnostic techniques for earlier diagnosis and more efficacious antibiotic selection, in addition, run more research regarding the patients’ comorbidities and the comorbidities’ effects on the VAP outcomes to establish more targeted and customized patients’ management.

Limitations

Our study is a retrospective study which makes it susceptible to the general limitations for retrospective studies including an inferior level of evidence compared with prospective studies, the risk of selection bias, determination of association only without causation, and the risk of unmeasured confounding. Moreover, the data collected from the medical record are always subjected to human coding errors and discrepancies of ICD coding among different hospitals. However, we tried to overcome these limitations by increasing our sample size to produce national estimates. We used a National Inpatient Sample (NIS) which is drawn from all states participating in HCUP, representing more than 95% of the United States population. This NIS approximates a 20% stratified sample of discharges from United States community hospitals [1].

## Conclusions

In our study, VAP in MV patients showed a downward trend from 2011 to 2016 with a small increase in the VAP incidence in the year 2017. Moreover, we found several significant independent predictors associated with VAP development such as young age, male gender, non-White race, large bed size hospitals, teaching hospitals, and several comorbidities including paralysis, weight loss, pulmonary circulatory disease, other neurological disorders, and electrolyte and fluid disorders. Finally, the LOS was noticeably higher in VAP patients throughout the study period, and the discharge to a facility was the most common outcome in VAP patients.
